# Altered microvasculature in pancreatic islets from subjects with type 1 diabetes

**DOI:** 10.1371/journal.pone.0276942

**Published:** 2022-10-31

**Authors:** Louise Granlund, Anders Hedin, Olle Korsgren, Oskar Skog, Marcus Lundberg

**Affiliations:** 1 Department of Immunology, Genetics and Pathology, Uppsala University, Uppsala, Sweden; 2 Department of Clinical Chemistry and Transfusion Medicine, Institute of Biomedicine, University of Gothenburg, Gothenburg, Sweden; Gifu University School of Medicine Graduate School of Medicine: Gifu Daigaku Igakubu Daigakuin Igakukei Kenkyuka, JAPAN

## Abstract

**Aims:**

The transcriptome of different dissociated pancreatic islet cells has been described in enzymatically isolated islets in both health and disease. However, the isolation, culturing, and dissociation procedures likely affect the transcriptome profiles, distorting the biological conclusions. The aim of the current study was to characterize the cells of the islets of Langerhans from subjects with and without type 1 diabetes in a way that reflects the *in vivo* situation to the highest possible extent.

**Methods:**

Islets were excised using laser capture microdissection directly from frozen pancreatic tissue sections obtained from organ donors with (n = 7) and without (n = 8) type 1 diabetes. Transcriptome analysis of excised samples was performed using AmpliSeq. Consecutive pancreatic sections were used to estimate the proportion of beta-, alpha-, and delta cells using immunofluorescence and to examine the presence of CD31 positive endothelial regions using immunohistochemistry.

**Results:**

The proportion of beta cells in islets from subjects with type 1 diabetes was reduced to 0% according to both the histological and transcriptome data, and several alterations in the transcriptome were derived from the loss of beta cells. In total, 473 differentially expressed genes were found in the islets from subjects with type 1 diabetes. Functional enrichment analysis showed that several of the most upregulated gene sets were related to vasculature and angiogenesis, and histologically, vascular density was increased in subjects with type 1 diabetes. Downregulated in type 1 diabetes islets was the gene set *epithelial mesenchymal transition*.

**Conclusion:**

A number of transcriptional alterations are present in islets from subjects with type 1 diabetes. In particular, several gene sets related to vasculature and angiogenesis are upregulated and there is an increased vascular density, suggesting an altered microvasculature in islets from subjects with type 1 diabetes. By studying pancreatic islets extracted directly from snap-frozen pancreatic tissue, this study reflects the *in vivo* situation to a high degree and gives important insights into islet pathophysiology in type 1 diabetes.

## Introduction

In type 1 diabetes (T1D) endogenous insulin-secretion is lost due to beta-cell destruction and affected subjects are dependent on exogenous insulin administration. Beta-cells make up the islet of Langerhans together with alpha-, delta-, PP- and ghrelin cells that are involved in glucose homeostasis, regulation of food intake and metabolism [1–3]. Several studies have been conducted in order to describe the different pancreatic cell types in subjects with [4, 5] and without diabetes [6–8]. However, in these studies the pancreatic islets have been obtained through a rough isolation process that separates them from their natural environment [9]. Although the islets remain functional and usable for transplantation [9], both the islet isolation process and culturing of islets affect the transcriptome of the cells [10, 11]. Furthermore, to analyze individual cells, the islets must be dissociated into single-cells and go through a cell-sorting process, likely increasing the risk of introducing alterations in the transcriptome which in the end may distort the biological conclusions.

Laser capture microdissection (LCM) ensures excision of islets directly from their natural environment–i.e. while still being encased in the pancreas. An advantage with this choice of method for tissue analysis is that the cells have not endured exposure to the islet isolation and dissociation process. By using LCM, the purpose of this study was to characterize islets in donors with a long duration of T1D and matched non-diabetic subjects, reflecting the *in vivo* situation to as high degree as possible, and thus enable uncovering of important transcriptional alterations.

## Materials and methods

### Human pancreatic specimens

Pancreases from heart-beating organ donors treated as for organ transplantation were procured through the Nordic Network for clinical Islet Transplantation (https://nordicislets.medscinet.com/en.aspx). Consent for organ donation was obtained verbally from the deceased’s next of kin by the attending physician and was documented in accordance with Swedish law and approved by the Regional Ethics Committee (DNR 2015/444). The pancreases were dissected, and biopsies were immediately snap-frozen in liquid nitrogen and subsequently stored at -80°C. At the time of the study, the biobank contained biopsies from 12 donors with long-standing T1D, and more than 2000 non-diabetic donors. Selection of biopsies, stainings, sectioning strategy and LCM was performed as described in Granlund *et*. *al*. [12]. Briefly, the donors with longstanding T1D were age-, sex- and BMI-matched to non-diabetic donors, and all biopsies were evaluated for immune infiltration by staining for CD45 and synaptophysin. Donors with a pronounced immune cell infiltration, or biopsies consisting mainly of fibrotic or adipose tissue were excluded from the study (described in Granlund *et*. *al*. [12]). Out of 24 donors examined (12 donors with long-standing T1D and 12 matched controls), 15 donors passed the screening and were included in the study. The characteristics of these are shown in Table 1.

**Table 1:** Clinical characteristics of the donors that passed the screening.

	Donor No.	Age	Sex	BMI (kg/m2)	HbA1c (mmol/mol)	Pancreas region frozen biopsies	IA2A	GADA	Cause of death
**NDs**	**ND-1**	13	M	16	40	tail	na	na	Celebral anoxia due to cardiac arrest
	**ND-2**	35	F	24.7	na	body	-	-	Hypoxemia due to cardiac arrest
	**ND-3**	57	F	22.7	45	tail	na	na	Subarachnoid hemorrage
	**ND-4**	45	F	25.4	38.8	tail	-	-	Infarction in the cerebellum
	**ND-5**	21	M	28	38.8	tail	-	-	Head trauma
	**ND-6**	17	F	28.9	na	body	na	na	Traumatic subarachnoid hemorrage
	**ND-7**	63	M	24	39.9	tail	-	-	Subarachnoid hemorrage
	**ND-8**	13	M	19.7	33	tail	-	-	Strangulation
**Mean**		**33**		**23.7**	**39.3**				
**T1Ds**	**T1D-1**	16	M	21.9	na	tail	na	na	Trauma subarachnoid hemorrage
	**T1D-2**	36	F	20.9	55.2	tail	na	na	Intracranial hemorrage
	**T1D-3**	60	F	23.9	66.1	tail	+	+	Subarachnoid hemorrage
	**T1D-4**	47	F	27.6	57.4	body	-	-	Cardiac arrest
	**T1D-5**	24	M	27.5	67.2	tail	+	-	Trauma
	**T1D-6**	25	F	26.7	54.1	tail	-	-	Cerebral edema due to hypoglycemia
	**T1D-7**	65	M	24.2	na	tail	na	na	Trauma by fall
**Mean**		**39**		**24.7**	**60**				

During the screening, donors with a pronounced immune cell infiltration, or biopsies consisting mainly of fibrotic or adipose tissue were excluded from the study. All islets in donors with T1D were insulin-negative. NDs: Non-diabetic subjects, T1Ds: Type 1 diabetic subjects. Na: not available. +: present, -: not present.

### Sectioning strategy of biopsies and immunofluorescent staining of endocrine cells

Frozen biopsies were sectioned and consecutive sections were alternately used for immunofluorescence (IF), LCM or immunohistochemistry (IHC), as described in Granlund *et al*. [12]. The sections intended for IF were stained for insulin, glucagon and somatostatin (S1 Table) and the slides were scanned on a confocal microscope (LSM700, Zeiss, Oberkochen, Germany) and used to locate islets that were microdissected on the consecutive PEN membranes. The methodology was designed to only extract and study insulin-negative islets in T1D subjects, but it was discovered that all islets were insulin-negative and therefore no selection of islets was necessary. To estimate the proportion of alpha, beta and delta cells, the cells of ten randomly chosen islets (in one section/donor) were annotated and the area in px^2^ was determined for alpha- beta- and delta cells respectively using the polygon tool in Qupath software (0.1.2). The area in px^2^ was converted to μm^2^ (0.8 pixels/μm). The average obtained by two independent investigators was calculated for each donor.

### Immunohistochemistry of endothelial cells

The consecutive sections were stained for CD31 and synaptophysin. Primary antibodies (S1 Table) were added and thereafter visualized using Dako EnVision Doublestain system (DAB+/Permanent Red). Sections were counterstained with hematoxylin (Histolab) and photographed using a Zeiss Palm Microbeam Ⅳ microscope at 20× magnification. The CD31 positive regions within islets was evaluated with assistance of ImageJ software. Ten islets per donor was analyzed; the islet area (μm^2^) and the length of all CD31 positive regions (μm) found within these islets were noted. The total length of CD31 positive regions per total islet area (vascular density) was calculated.

### Laser capture microdissection (LCM)

LCM was performed as described in Granlund *et al*. [12]. In brief, the frozen sections were thawed and dehydrated after which they were mounted on an Arcturus XT LCM system (Thermo Fisher Scientific, Massachusetts). Islets were identified based on islet auto-fluorescence and verified by the scanned IF slides. The islets were captured on an Arcturus CapSure HS LCM Cap (LCM0215, Thermo Fisher Scientific, Massachusetts) and incubated in 1% beta-Mercaptoethanol in a heating block for 30 min at 42°C, lysing the tissue. The lysates were stored at -80°C until RNA extraction. Areas of the cut regions were noted and the diameter of the islets calculated according to ($(\surd \frac{Area}{\pi })\times 2=diameter$, S1 Fig).

### RNA extraction and transcriptome analysis

The samples were brought to room temperature by short incubation at 37°C. All LCM extracted samples were pooled for each donor. RNA was extracted with the RNeasy Plus Micro kit (Qiagen, Sweden) according to the manufacturer’s protocol for purification of total RNA from microdissected cryosections. Samples were eluted with RNase-free water and stored at -80°C until transcriptome analysis, which was performed using the Ion AmpliSeq Transcriptome Gene Expression Kit (Thermo Fisher Scientific, Massachusetts) and sequencing on an IonS5XL instrument, as described previously in detail [12]. Acquired reads were analyzed using the ampliSeqRNA plugin in the Torrent Suite Server version 5.10.1. The reads were aligned to hg19 AmpliSeq Transcriptome ERCC v1, quantifying expression data for 20,813 genes.

### Statistical analysis

#### Filtering

Data was analyzed with R (v. 4.2.1) in Rstudio (v. 22.02.3) using the edgeR R package (v. 3.36.0) [13, 14] starting from raw read counts. As the islet samples were extracted, prepared and sequenced together with several samples from the exocrine portion of the pancreas, as described in more detail previously [12], the exocrine libraries were not excluded in the current data analysis. I.e. the exocrine libraries were included when creating the DGElistobject, as well as subsequent filtering, normalization and creation of the generalized linear model. Genes with more than 10 counts per million (CPM) in at least 6 samples were retained using the filterByExp function of edgeR.

#### Deconvolution analysis

Cell type proportions of the LCM extracted bulk data was estimated with Multi-subject Single Cell (MuSiC) deconvolution using the R package MuSiC(v. 0.2.0) [15]. The raw counts were analyzed with the E-MTAB-5061 human pancreas single-cell data as reference dataset (https://www.ebi.ac.uk/arrayexpress/experiments/E-MTAB-5061/) [7]. Results were visualized using ggplot2 (v 3.3.0).

#### Normalization

Raw count normalization was performed using the trimmed mean of M values (TMM) [16] method with the calcNormFactors- function of EdgeR.

#### Data structure

TMM- adjusted and log- normalized counts were used to visualize the data structure by principal component analysis (PCA) using the R- package PCAtools(v.2.6.0) [17].

#### Differential gene expression analysis

Differentially expressed genes (DEGs) between islets from subjects with and without T1D were analyzed using a generalized linear model and a quasi-likelihood test with the glmQLFit and glmTreat functions of edgeR. Genes differentially expressed in T1D compared with non-diabetic subjects were assessed. FDR-adjusted P-values were calculated using the Benjamini- Hochberg method in the topTags function in edgeR. Criteria for differential expression was FDR-adjusted P-value less than 0.05 while testing for an absolute log fold change ≥ log2(1.2).

#### Functional enrichment—and overrepresentation analysis

Competitive gene set testing was conducted with CAMERA (Correlation Adjusted MEan RAnk test) [18] using the CAMERA function in edgeR [13, 14] and the MSigDB Hallmark set [19]. A gene set was considered significantly enriched if the FDR- adjusted p-value was <0.25. Overrepresentation analysis (ORA) was done using a hypergeometric test [20, 21]. The ORA was performed by testing the DEG (FC± log2(1.2), FDR>0.05) against gene ontology: biological processes (GO:BP) [22, 23] and REACTOME [24, 25] terms using the function g:GOSt in g:Profiler [26]. The g:SCS corrected P- values <0.05 were required for a set to be considered significantly enriched.

#### Comparison of vascular density in islets

The Mann–Whitney test was used in GraphPad Prism software (version 6.0h) to compare the islet vascular density in non-diabetic and T1D subjects. A p-value < 0.05 was considered statistically significant.

## Results

### The proportion of different endocrine cells was similar in histological and transcriptional data

**Fig 1:**
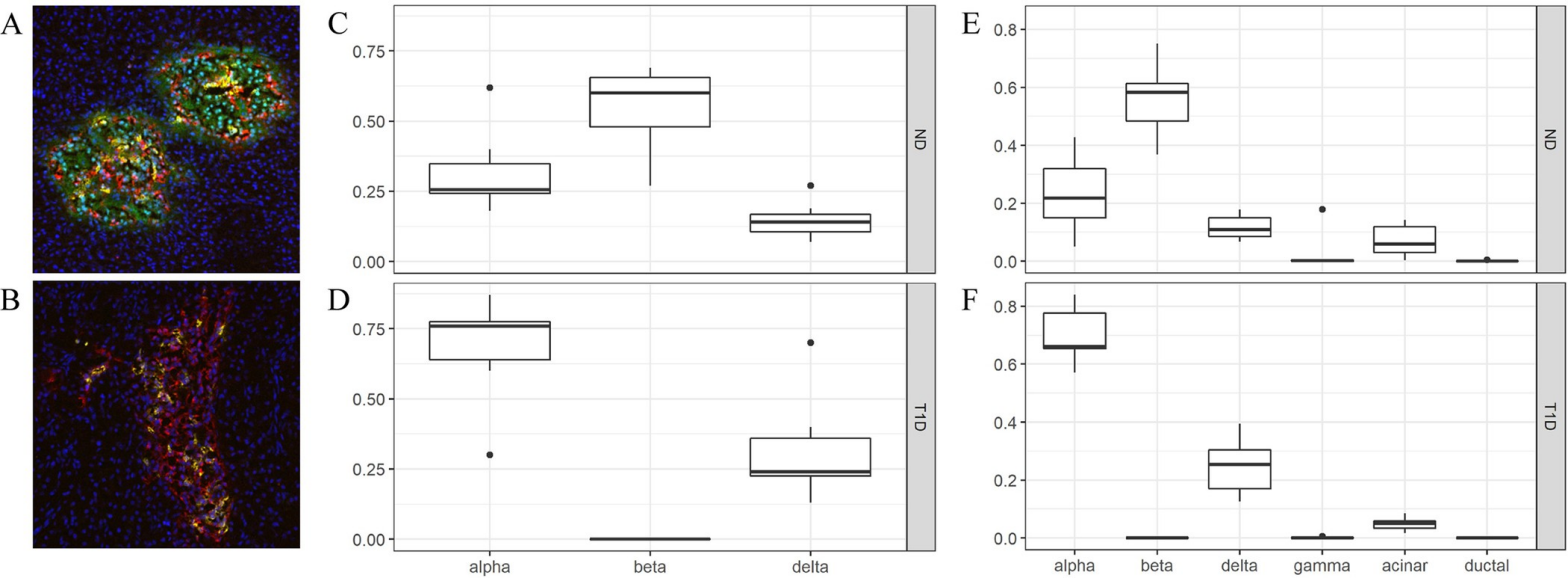
The proportion of different endocrine cells in islets. Images of representative islets, where the composition of islet cells based on immunofluorescent triple-staining of glucagon (red), insulin (green) and somatostatin (yellow) to represent alpha-, beta- and delta cells respectively, is shown in non-diabetic subjects (A) and T1D subjects (B). Tukey box-plots of the histologically determined endocrine area proportion in non-diabetic subjects (C) and T1D subjects (D). Proportions of the area sum to one per sample. Multi-subject Single Cell (MuSiC) utilizes cell-type specific gene expression from single-cell RNA sequencing data to characterize cell type compositions from bulk data. Deconvolution of the bulk data into alpha, beta, gamma, delta, acinar and ductal cells in the different tissues is illustrated in non-diabetic subjects (E) and T1D subjects (F). Proportions sum to one per sample and the data is illustrated in a Tukey boxplot. ND: Non-diabetic subjects, T1D: Type 1 diabetic subjects.

The median endocrine area proportion in non-diabetic subjects was histologically determined to be 26% alpha cells, 60% beta cells and 14% delta cells (Fig 1A and 1C). The median endocrine area proportion in subjects with T1D was 76% alpha cells, 0% beta cells and 24% delta cells (Fig 1B and 1D). Based on estimation from the transcriptome data through a Multi-subject Single Cell (MuSiC) analysis (Fig 1E and 1F), the proportion of the different endocrine cells was similar to the histologically estimated endocrine area proportion in non-diabetic and T1D pancreases. Islet tissue extracted by LCM had only limited contamination of exocrine tissue (acinar median 0.059%, and 0.050%, and ductal median 0% and 0% in tissue extracted from pancreases of non-diabetic and T1D subjects respectively) according to the MuSiC analysis (Fig 1E and 1F).

### Gene sets related to vasculature and angiogenesis were upregulated in islets from subjects with T1D and the gene set epithelial mesenchymal transition was downregulated

**Fig 2:**
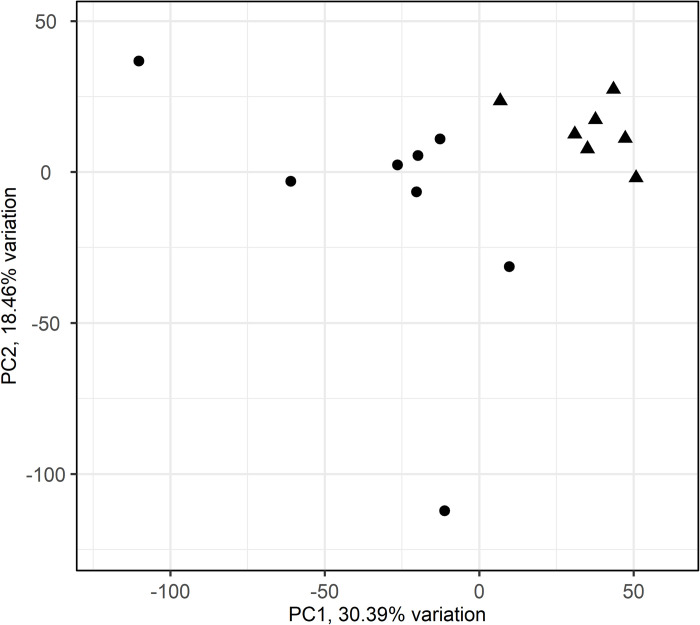
Principal component analysis (PCA)–islets from subjects with and without type 1 diabetes clusters separately. The 25% most variable genes of islet were used for PCA. Each point corresponds to a sample plotted by PC1 and PC2. PC1 and PC2 describe 30.4% and 18.5% of the islet variation, respectively. Circles = Non-diabetic subjects, triangles = Type 1 diabetic subjects.

**Fig 3:**
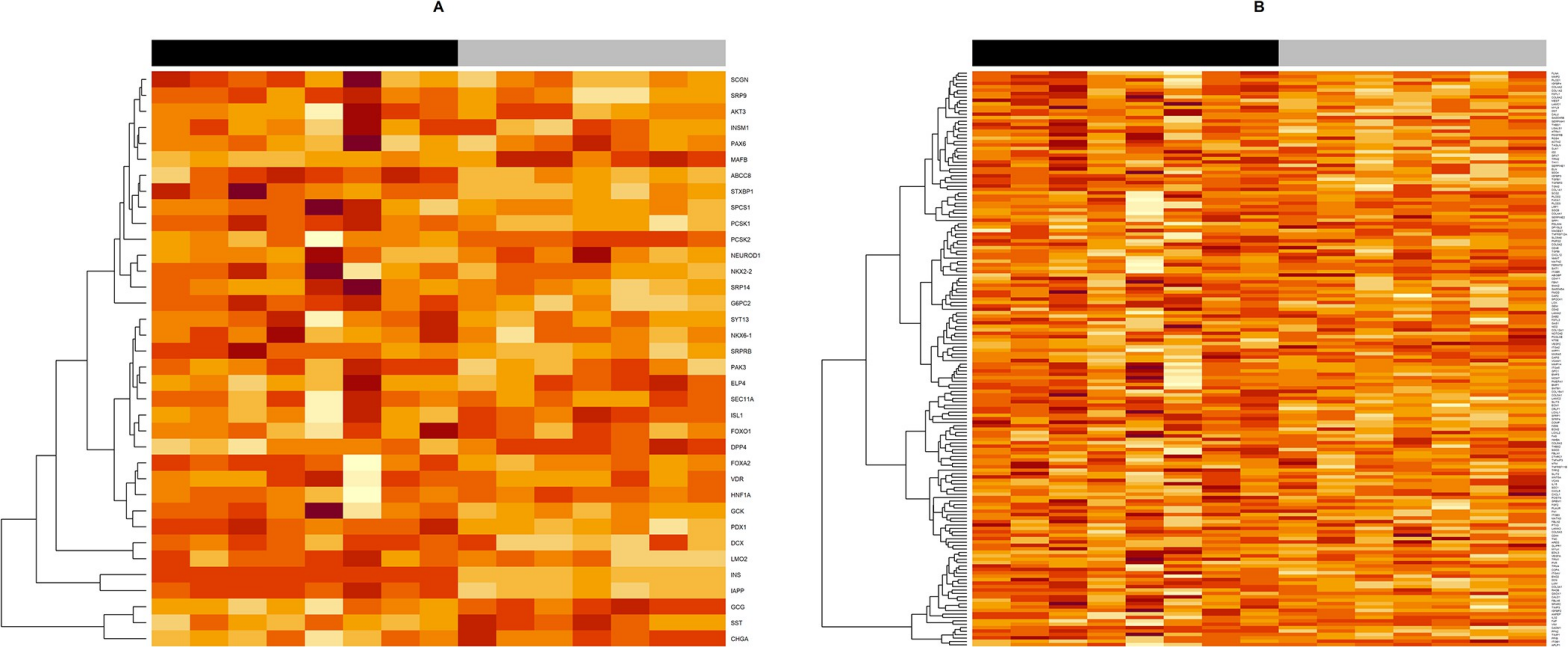
Heatmap of gene sets significantly altered in type 1 diabetes using CAMERA (Correlation Adjusted MEan RAnk test). Using CAMERA and the MSigDB Hallmark gene set collection, a gene set was considered significantly enriched if the FDR- adjusted p-value was <0.25. Two gene sets were found to be downregulated, and the expression of all the genes in the gene set is illustrated in (A) *Hallmark Pancreas Beta Cells* and (B) *Hallmark Epithelial Mesenchymal Transition*. Darker colour indicates higher expression. Black = Non-diabetic subjects, Grey = Type 1 diabetic subjects.

A PCA of the 25% most variable genes across islets shows that islets from T1D and non-diabetes subjects clustered separately (Fig 2). The differential gene expression analysis revealed 347 DEGs that were downregulated and 126 that were upregulated in islets from subjects with T1D (S1 File). Overrepresentation analysis with g:Profiler of these DEGs identified many gene sets of diverse functions to be upregulated in T1D (g:SCS adjusted p-value <0.05) (S1 File). The top 10 enriched gene sets using Gene Ontologi: Biological Processes (GO:BP) were mostly related to vasculature, angiogenesis and anatomical structuring, whereas many of the top 10 gene sets using REACTOME were associated with insulin signalling (Table 2A). The gene sets that were downregulated in T1D (g:SCS adjusted p-value <0.05) were mainly related to synaptic- and cell signalling as well as beta-cell loss (Table 2B and S1 File). Competitive gene set testing with CAMERA, and using the MSigDB Hallmark gene set collection, showed that *pancreas beta cells* (Fig 3A) and *epithelial mesenchymal transition* (Fig 3B) were downregulated in islets from subjects with T1D.

**Table 2:** Top 10 enriched gene sets using g:Profiler.

**A**	
**Upregulated gene sets in islets from subjects with T1D**	
**Gene Ontology: Biological Processes**	**Adjusted P-value**
Anatomical structure morphogenesis	2.090×10^−6^
Blood vessel morphogenesis	9.070×10^−6^
Angiogenesis	3.769×10^−5^
Regulation of developmental process	6.611×10^−5^
Blood vessel development	6.953×10^−5^
Regulation of cellular process	9.713×10^−5^
Vasculature development	1.335×10^−4^
Circulatory system development	1.572×10^−4^
Anatomical structure development	1.909×10^−4^
Anatomical structure formation involved in morphogenesis	2.708×10^−4^
**Reactome**	**Adjusted P-value**
IRS-mediated signalling	4.972×10^−3^
Signaling by PDGFR in disease	6.897×10^−3^
IRS-related events triggered by IGF1R	8.108×10^−3^
IGF1R signaling cascade	8.108×10^−3^
Signaling by Type 1 Insulin-like Growth Factor 1 Receptor (IGF1R)	9.090×10–3
Downstream signaling of activated FGFR1	9.200×10^−3^
Signaling by FGFR3 fusions in cancer	9.906×10^−3^
Insulin receptor signalling cascade	1.016×10^−2^
Downstream signaling of activated FGFR2	1.107×10^−2^
Biogenic amines are oxidatively deaminated to aldehydes by MAOA and MAOB	1.370×10^−2^
**B**	
**Downregulated gene sets in islets from subjects with T1D**	
**Gene Ontology: Biological Processes**	**Adjusted P-value**
Cell-cell signaling	1.653×10^−7^
Nervous system development	4.565×10^−6^
System development	6.374×10^−5^
Multicellular organism development	1.171×10^−4^
Multicellular organismal process	2.554×10^−4^
Regulation of cell communication	3.267×10^−4^
Anterograde trans-synaptic signaling	3.936×10^−4^
Chemical synaptic transmission	3.936×10^−4^
Signaling	4.121×10^−4^
Trans-synaptic signaling	4.545×10^−4^
**Reactome**	**Adjusted P-value**
Regulation of gene expression in beta cells	1.108×10^−4^
Regulation of beta-cell development	1.038×10^−3^
Amyloid fiber formation	4.418×10^−2^

347 differentially expressed genes (DEGs) were found to be downregulated and 126 were upregulated in islets from subjects with T1D. Overrepresentation analysis on the DEGs was done using g:Profiler. Top 10 upregulated gene sets in donors with type 1 diabetes using Gene Ontology: biological processes and REACTOME is shown in (A). Top 10 downregulated gene sets in donors with type 1 diabetes using Gene Ontology: biological processes and REACTOME is shown in (B). Padj: Adjusted p-value according to g:SCS.

### Vascular density in islets was increased in subjects with T1D

**Fig 4:**
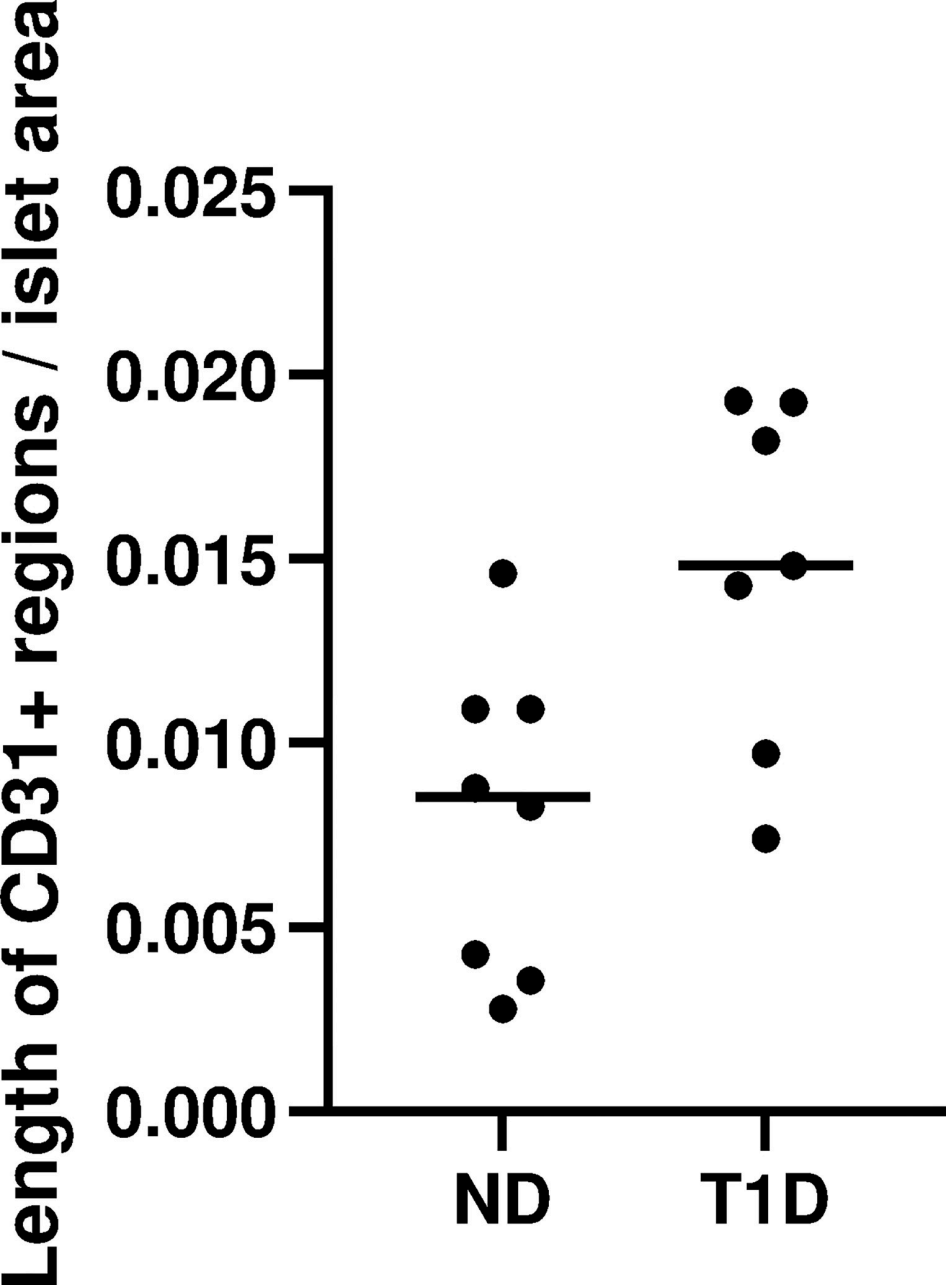
Vascular density (length of CD31^+^ regions [μm] per islet area [μm^2^]). The total length of CD31 positive regions within islets, were divided by the total islet area. ND = non-diabetic samples, T1D = type 1 diabetic samples.

CD31 staining revealed an increased total endothelial length per total islet area (vascular density) in subjects with T1D compared with non-diabetic subjects (median total endothelial length per total islet area was 0.015 μm/μm^2^ and 0.0085 respectively) (p = 0.0263) (Fig 4). When excluding CD31^+^ regions shorter than 15 μm to account for the presence of possible individual macrophages, the vascular density in subjects with T1D was also increased (p = 0.0263).

## Discussion

In the current study, LCM was used to microdissect and analyze islets from subjects with T1D directly from frozen well-preserved pancreatic tissue obtained from heart-beating organ donors. As such, artefacts induced by enzymatic islet isolation, dispersion into single-cells, and culture were avoided, making the analysed transcriptomes to the highest possible extent reflect the *in vivo* situation. A hallmark of islets from subjects with T1D is the loss of beta cells. The islets examined in the current study were devoid of beta cells as determined both by a histological and transcriptional evaluation of the islet composition. Transcriptional alterations likely derived from the beta cell loss was reflected in the data regardless of bioinformatical approach; the beta cell associated genes insulin, MAFA and PDX1, were found among the downregulated DEGs, ORA of the downregulated DEGs with REACTOME showed gene sets such as *Regulation of gene expression in beta cells*, and using competitive gene set testing with CAMERA, the gene set *pancreas beta cells* was found to be downregulated. As the beta-cell loss correlates with expected transcriptional alterations, this finding suggests that the methodology is sound.

In total, 473 DEGs were discovered in the islets from subjects with T1D. Among these, the top 5 upregulated genes; *CIDEC*, *RSPO3*, *SLITRK6*, *TPD52L1* and *FGF10*, have unclear roles in islets. Interestingly, ORA of the DEGs using Gene Ontology: Biological Processes showed that the top 10 enriched gene sets in islets from subjects with T1D were mostly related to vasculature and angiogenesis. Neither these gene sets, nor versions of them, have been reported to distinguish different pancreatic cell types from each other in single-cell studies [5, 6], suggesting that these transcriptional alterations of the islets from subjects with T1D were not derived solely from the loss of beta cells. In support of the notion that the alterations seen in gene sets related to microvasculature in our study were not an effect of the beta-cell loss, these gene sets were not found to vary between different sorted islet cell types when running an ORA on the DEGs reported by Muraro *et al*. [6], (S2 File). However, gene sets related to anatomical structuring were upregulated in sorted alpha- and delta cells compared to other pancreatic cells [6] (S2 File), which likely explains the upregulation of these gene sets in the islets from subjects with T1D in our study.

In sections consecutive to those used for LCM, the vascular density (total CD31^+^ endothelial length per total islet area) was increased in T1D subjects. CD31 is also present on macrophages, but during the screening procedure, samples with a pronounced immune infiltration were excluded. Additionally, gene sets related to inflammation were not present among the upregulated gene sets, and a macrophage marker, CD68, was not present among the DEGs. This indicates that the elevated vascular density in T1D subjects was caused by an increased presence of endothelial cells. Similarly, a previous histological study reported an increase in the number of islet vessels, but with a reduced diameter [27]. The presence of upregulated gene sets related to angiogenesis as well as elevated vascular density suggests active angiogenesis and vascular remodelling in islets from subjects with T1D. Importantly, islet size is not altered in subjects with T1D despite the near total loss of beta cells [28], suggesting the increased vascular density to be due to an absolute increase in islet microvasculature. The islet endothelial cells play a crucial role in islet function and have been shown to both stimulate insulin secretion and play a role in beta cell function and proliferation [29–32]. The upregulation of gene sets related to the microvasculature may suggest an effort towards beta cell differentiation to compensate for the loss of beta cells. Another interpretation of the upregulation of genes related to microvasculature, is that it is a response to a disturbance in islet blood perfusion. If there is an insufficient blood circulation to the islet, this could contribute to the alpha-cell dysfunction reported in T1D [4, 33, 34].

There is no transcriptional variation between microvascular endothelial cells sorted from donors with or without impaired glucose metabolism (IGM) [35]. This suggests that the islet endothelial transcriptome is not per se affected as a consequence of diabetes, i.e. hyperglycemia, which mean that alterations seen in the islet microvasculature could instead be a contributing cause of T1D. However, the endothelial cells in islets from subjects with T1D could also be more severely affected than in subjects with IGM. Alternatively, differences in this cell type are only observable for study in intact, LCM-excised, islets. Indeed, microvascular epithelial cells are especially sensitive to the islet isolation process and culturing [36, 37].

Epithelial mesenchymal transition (EMT) is a biological process where epithelial cells are transitioned to mesenchymal cells, and is, among other things, important during embryogenesis and organ development as well as wound healing [38]. However, even fully differentiated epithelium can change its phenotype through activation of EMT. This enables transdifferentiation of epithelial cells to mesenchymal derivates even during adulthood, and has been shown to occur in the adult human exocrine pancreas [39, 40]. Through this process, EMT has been shown to be involved in beta cell differentiation and islet formation. Individual beta cells become insulin-positive in the progenitor epithelium, after which they lose epithelial characteristics and migrate out of the epithelial layer to form islets. As beta cells exit the epithelial progenitor cell layer, they acquire mesenchymal characteristics [41]. Vimetin, a mesenchymal cell marker, has been found in adult human islets, indicating that even mature islets might have a plasticity, which could require a mesenchymal phenotype [41]. Using competitive gene set testing with CAMERA, the gene set *epithelial mesenchymal transition* was found to be downregulated in islets from subjects with T1D. This could suggest that islets from donors with T1D have a lower degree of plasticity, and a less active EMT, however we cannot exclude that this is merely an effect of the lost beta cells.

In summary, a large number of transcriptional alterations were discovered in intact LCM-excised islets from subjects with T1D. Although many of these alterations likely are an effect of comparing islets devoid of beta cells in T1D with islets dominated by beta cells in non-diabetic controls, some of the discovered alterations emerge as potentially important for understanding the pathogenesis of the disease. Among these, there was an upregulation of several gene sets related to vasculature and angiogenesis, as well as an increased vascular density, demonstrating microvasculature to be altered in T1D. By studying pancreatic islets directly procured from frozen pancreatic sections, this study minimizes artifacts induced by handling the cells and uncovers potentially relevant insights into the pathophysiology of T1D.

## Supporting information

S1 Table Antibodies and stain used for immunofluorescence staining and immunohistochemistry. (DOCX)

S1 Fig Diameter of excised islets. Excised islet area was converted to diameter according to ((√*Area/π*)×2 = *diameter*). The proportion of islets within 50 μm intervals is illustrated in the histogram. T1D = type 1 diabetic samples, ND = non-diabetic samples.
(TIF)

S1 File Lists of differentially expressed genes and overrepresentation analysis results. Lists of the 347 DEGs that were downregulated, and the 126 DEGs that were upregulated in T1D, as well as the results from the overrepresentation analysis done with g:Profiler.
(XLSX)

S2 File The differential gene expression of alpha-, beta-, delta-, pp- and epsilon- cells reported by Muraro *et al*. [6], as well as the results of the overrepresentation analysis. To make sure that the enriched gene sets in islets from subjects with T1D, which were mostly related to vasculature and angiogenesis, were not derived solely from the loss of beta cells, an ORA using g:Profiler was done on a previously published data set as a reference [6]. DEGs of the different islet cell types reported by Muraro *et al*. did not show a link to gene sets related to vasculature and angiogenesis.
(XLSX)
