# Supplementary material for: Altered microvasculature in pancreatic islets from subjects with type 1 diabetes
Source: PLoS One. 2022 Oct 31;17(10):e0276942. doi: 10.1371/journal.pone.0276942 (PMC9621430; doi:10.1371/journal.pone.0276942)
Supplement: S1 Table — (DOCX) [file pone.0276942.s001.docx]

| **Antibody** | **Catalog #** | **Company** | **Concentration** | **Incubation time** |
| --- | --- | --- | --- | --- |
| **Insulin** | 565689 | BD Biosciences | 1:100 | 1 h |
| **Glucagon** | 565891 | BD Biosciences | 1:100 | 1 h |
| **Somatostatin** | 566032 | BD Biosciences | 1:100 | 1 h |
| **Sytox Orange** | S11368 | Life Technologies | 500 nM | 10 min |
| **CD31** | 303101 | BioLegend | 1:500 | 30 min |
| **Synaptophysin** | GA66061-2 | Agilent | 1:100 | 30 min |

**S1 Table.** Antibodies and stain used for immunofluorescence staining and immunohistochemistry.
