# Supplementary figures and images for: Altered microvasculature in pancreatic islets from subjects with type 1 diabetes
Source: PLoS One. 2022 Oct 31;17(10):e0276942. doi: 10.1371/journal.pone.0276942 (PMC9621430; doi:10.1371/journal.pone.0276942)

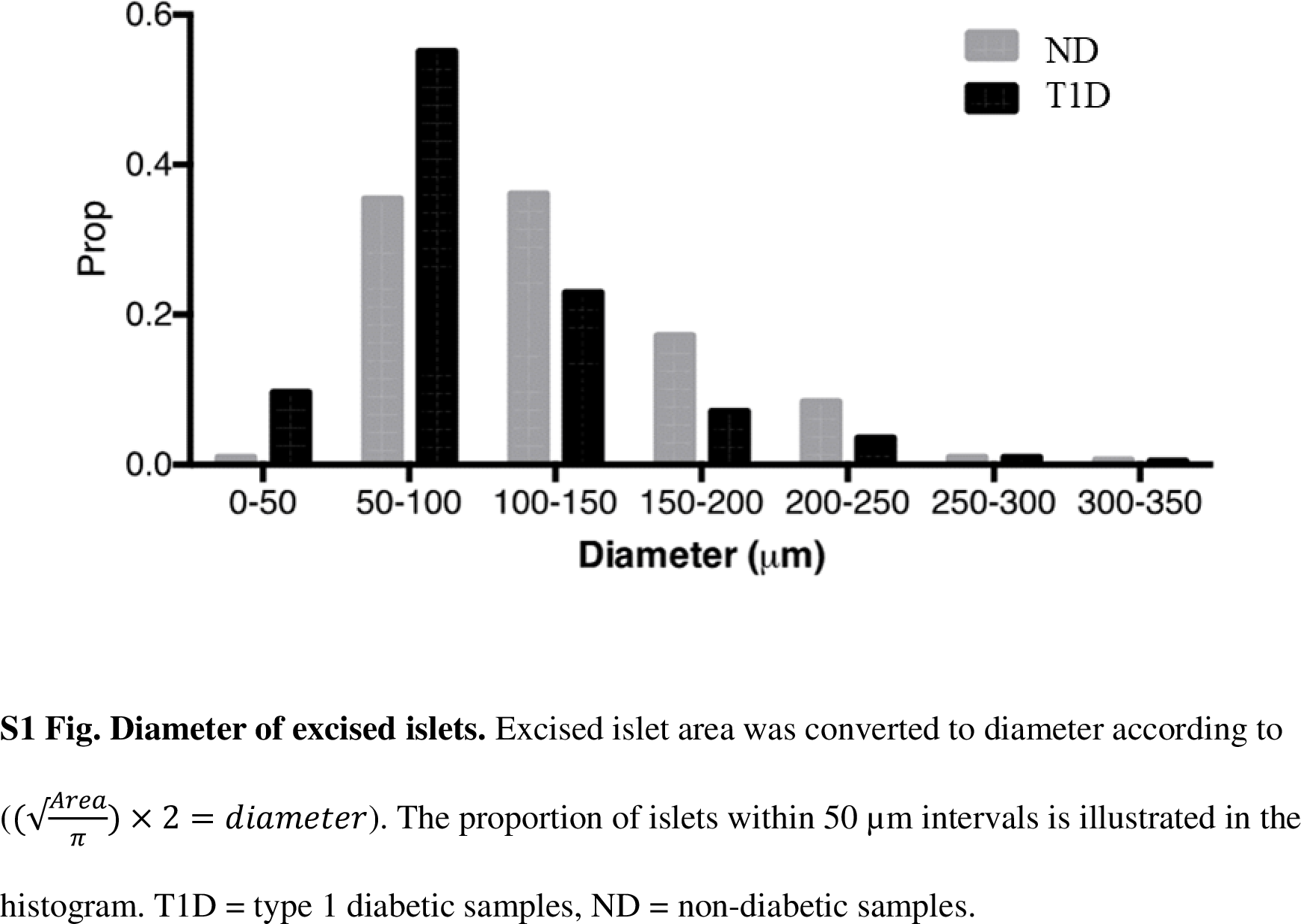

Supplement: S1 Fig — Excised islet area was converted to diameter according to ((√Area/π)×2 = diameter). The proportion of islets within 50 μm intervals is illustrated in the histogram. T1D = type 1 diabetic samples, ND = non-diabetic samples. (TIF) [file pone.0276942.s002.tif]
